# Supplementary material for: All-optical information-processing capacity of diffractive surfaces
Source: Light Sci Appl. 2021 Jan 28;10:25. doi: 10.1038/s41377-020-00439-9 (PMC7844294; doi:10.1038/s41377-020-00439-9)
Supplement: Supplementary file 1 — Supplementary Information [file 41377_2020_439_MOESM1_ESM.pdf]

Supplementary Information for

All-Optical Information Processing Capacity of Diffractive Surfaces

Onur Kulce[1,2,3,§], Deniz Mengu[1,2,3,§], Yair Rivenson[1,2,3] , Aydogan Ozcan[1,2,3,*]

[1] Electrical and Computer Engineering Department, University of California, Los Angeles, CA, 90095, USA

[2] Bioengineering Department, University of California, Los Angeles, CA, 90095, USA

[3] California NanoSystems Institute, University of California, Los Angeles, CA, 90095, USA

[§] Equal contribution

* Corresponding author: ozcan@ucla.edu

S1. Coefficient and basis vector generation algorithm for an optical network formed by two diffractive surfaces: Special case for $N_{L1} = N_{L2} = N_i = N_o = N$

Here we present a coefficient and basis vector generation algorithm, which is given by Table S1, specific for an optical network formed by two equally-sized diffractive surfaces and input/output fields-of-view, i.e., $N_{L1} = N_{L2} = N_i = N_o = N$. The presented algorithm is a special case of the algorithm reported in Table 1 of the main text, when the chunk partition is $n_1 = n_2 = \cdots = n_s = 1$. So, this analysis further confirms the fact that the solution space created by a 2-layered diffractive network forms a $(2N-1)$-dimensional subspace of an $N_i N_o = N^2$-dimensional vector space.

For the special case of $N_{L1} = N_{L2} = N_i = N_o = N$, $\boldsymbol{a_2}$, given by Equation 6 of the main text, can be written as:

$$\boldsymbol{a_2} = \sum_{i=1}^{2N-1} c_i \boldsymbol{b_i} \quad (S1)$$

where c_i and $\boldsymbol{b_i}$ are an arbitrary complex-valued coefficient and the corresponding basis vector, respectively. These are reported in the last column of Table S1 such that $\boldsymbol{b_i}$ is the term that remains inside the parenthesis and c_i is the associated coefficient. As can be seen from Table S1, the coefficient, $t_{1,i}$ or $t_{2,j}$ for $i, j \in \{1,2,\ldots,N\}$, can be chosen independently in each step. It is also straightforward to show that all the N^2-many $\boldsymbol{h_{ij}}$ vectors are consumed in the generation of new basis functions by computing the summations of the used $\boldsymbol{h_{ij}}$ vectors at each step. Also, without loss of generality, the chosen transmittance index and the corresponding $\boldsymbol{h_{ij}}$ in each step may change. This ends up with a different basis vector $\boldsymbol{b_i}$, but the dimensionality of the solution space does not change.

Step	Choice from $\boldsymbol{T_1}$	Choice from $\boldsymbol{T}_2$	Corresponding Basis Vectors $\boldsymbol{h_{ij}}$	Resulting Coefficient and Basis Vector
1	$t_{1,1}$	$t_{2,1}$ (fixed)	$\boldsymbol{h_{11}}$	$c_1\boldsymbol{b_1} = t_{1,1}(t_{2,1}\boldsymbol{h_{11}})$
2	-	$t_{2,2}$	$\boldsymbol{h_{12}}$	$c_2\boldsymbol{b_2} = t_{2,2}(t_{1,1}\boldsymbol{h_{12}})$
3	$t_{1,2}$	-	$\boldsymbol{h_{21}}, \boldsymbol{h_{22}}$	$c_3\boldsymbol{b_3} = t_{1,2}(t_{2,1}\boldsymbol{h_{21}} + t_{2,2}\boldsymbol{h_{22}})$
4	-	$t_{2,3}$	$\boldsymbol{h_{13}}, \boldsymbol{h_{23}}$	$c_4\boldsymbol{b_4} = t_{2,3}(t_{1,1}\boldsymbol{h_{13}} + t_{1,2}\boldsymbol{h_{23}})$
5	$t_{1,3}$	-	$\boldsymbol{h_{31}}, \boldsymbol{h_{32}}, \boldsymbol{h_{33}}$	$c_5\boldsymbol{b_5} = t_{1,3}(t_{2,1}\boldsymbol{h_{31}} + t_{2,2}\boldsymbol{h_{32}} + t_{2,3}\boldsymbol{h_{33}})$
6	-	$t_{2,4}$	$\boldsymbol{h_{14}}, \boldsymbol{h_{24}}, \boldsymbol{h_{34}}$	$c_6\boldsymbol{b_6} = t_{2,4}(t_{1,1}\boldsymbol{h_{14}} + t_{1,2}\boldsymbol{h_{24}} + t_{1,3}\boldsymbol{h_{34}})$
⋮	⋮	⋮	⋮	⋮
2n-2	-	$t_{2,n}$	$\boldsymbol{h_{1n}}, \boldsymbol{h_{2n}}, \cdots$ $\boldsymbol{h_{(n-2)n}}, \boldsymbol{h_{(n-1)n}}$	$c_{2n-2}\boldsymbol{b_{2n-2}} = t_{2,\mathrm{n}}\left(\sum_{i=1}^{n-1} t_{1,i}\boldsymbol{h_{in}}\right)$
2n-1	$t_{1,n}$	-	$\boldsymbol{h_{n1}}, \boldsymbol{h_{n2}}, \cdots$ $\boldsymbol{h_{n(n-1)}}, \boldsymbol{h_{nn}}$	$c_{2n-1}\boldsymbol{b_{2n-1}} = t_{1,n}\left(\sum_{i=1}^{n} t_{2,i}\boldsymbol{h_{ni}}\right)$
⋮	⋮	⋮	⋮	⋮
2N-2	-	$t_{2,N}$	$\boldsymbol{h_{1N}}, \boldsymbol{h_{2N}}, \cdots$ $\boldsymbol{h_{(N-2)N}}, \boldsymbol{h_{(N-1)N}}$	$c_{2N-2}\boldsymbol{b_{2N-2}} = t_{2,\mathrm{N}}\left(\sum_{i=1}^{N-1} t_{1,i}\boldsymbol{h_{iN}}\right)$
2N-1	$t_{1,N}$	-	$\boldsymbol{h_{N1}}, \boldsymbol{h_{N2}}, \cdots$ $\boldsymbol{h_{N(N-1)}}, \boldsymbol{h_{NN}}$	$c_{2N-1}\boldsymbol{b_{2N-1}} = t_{1,N}\left(\sum_{i=1}^{N} t_{2,i}\boldsymbol{h_{Ni}}\right)$

Table S1. *Coefficient and basis vector generation algorithm for a 2-layered diffractive network (K = 2) when* $N_{L1} = N_{L2} = N_i = N_o = N$

S2. Coefficient and basis vector generation algorithm for an optical network formed by three diffractive surfaces: Special case for $N_{L1} = N_{L2} = N_{L3} = N_i = N_o = N$

In the subsection of the main text titled "*Analysis of an optical network formed by three or more diffractive surfaces*", we showed that a network having equally-sized three diffractive layers and input/output fields-of-views can cover $(3N-2)$-dimensional subspace of an N^2-dimensional vector space, where the proof was based on the addition of a third diffractive layer that has size N to an existing diffractive network that has two surfaces, presenting a dimensionality of $(2N-1)$. In this subsection we demonstrate the same conclusion by directly employing a diffractive network that has equally-sized three diffractive surfaces, i.e., $N_{L1} = N_{L2} = N_{L3} = N_i = N_o = N$.

The input-output relation for a network that has three diffractive surfaces can be written as:

$$\boldsymbol{y} = \boldsymbol{H_{d_4}T_3H_{d_3}T_2H_{d_2}T_1H_{d_1}x} = \boldsymbol{A_3x} \qquad S2$$

where $\boldsymbol{H_{d_i}}$ for $i \in \{1,2,3,4\}$ are $N \times N$ matrices which represent the free-space propagation, $\boldsymbol{T_i}$ for $i \in \{1,2,3\}$ are $N \times N$ matrices which represent the multiplicative effect of the ith diffractive

surface, and $\boldsymbol{x}$ and $\boldsymbol{y}$ are length-N vectors which represent the input and output fields-of-view, respectively. Details of these matrices and vectors can be found in "*Theoretical analysis of the information-processing capacity of diffractive surfaces*" subsection of the main text. In this subsection, we show that $vec(\boldsymbol{A_3}) = \boldsymbol{a_3}$ can be written as:

$$\boldsymbol{a_3} = \sum_{i=1}^{3N-2} c_i \boldsymbol{b_i} \qquad S3$$

where c_i and $\boldsymbol{b_i}$ are an arbitrary complex-valued coefficient and the corresponding basis vector, respectively. We first perform the vectorization of $\boldsymbol{A_3}$ as:

$$\begin{aligned} vec(\boldsymbol{A_3}) = \boldsymbol{a_3} &= vec\big(\boldsymbol{H_{d_4}T_3H_{d_3}T_2H_{d_2}T_1H_{d_1}}\big) \\ &= \big(\boldsymbol{H_{d_1}^T} \otimes \boldsymbol{H_{d_4}}\big)(\boldsymbol{T_1^T} \otimes \boldsymbol{T}_3)\big(\boldsymbol{H_{d_2}^T} \otimes \boldsymbol{H_{d_3}}\big)vec(\boldsymbol{T_2}) \\ &= \big(\boldsymbol{H_{d_1}^T} \otimes \boldsymbol{H_{d_4}}\big)(\boldsymbol{T_1} \otimes \boldsymbol{T}_3)\big(\boldsymbol{H_{d_2}^T} \otimes \boldsymbol{H_{d_3}}\big)vec(\boldsymbol{T_2}) \end{aligned} \qquad S4$$

In order to uncouple the transmittance and free-space propagation parts in Equation S4, we define a matrix $\widehat{\boldsymbol{H}}_{\boldsymbol{d_{23}}}$ having size $N^2 \times N^4$ from $\boldsymbol{H_{d_2}^T} \otimes \boldsymbol{H_{d_3}}$ as:

$$\widehat{\boldsymbol{H}}_{\boldsymbol{d_{23}}} = \begin{bmatrix} \boldsymbol{r_1} & \boldsymbol{0} & \boldsymbol{0} & \cdots & \boldsymbol{0} \\ \boldsymbol{0} & \boldsymbol{r_2} & \boldsymbol{0} & \cdots & \boldsymbol{0} \\ \boldsymbol{0} & \boldsymbol{0} & \boldsymbol{r_3} & \cdots & \boldsymbol{0} \\ \vdots & \vdots & \vdots & \vdots & \vdots \\ \boldsymbol{0} & \boldsymbol{0} & \boldsymbol{0} & \cdots & \boldsymbol{r_{N^2}} \end{bmatrix} \qquad S5$$

where $\boldsymbol{r_i}$ is the ith row of $\boldsymbol{H_{d_2}^T} \otimes \boldsymbol{H_{d_3}}$ and $\boldsymbol{0}$ is an all-zero row vector with size $1 \times N^2$. Therefore, in each row of $\widehat{\boldsymbol{H}}_{\boldsymbol{d_{23}}}$, there are $N^4 - N^2$ many zeros. Also, let $\boldsymbol{t_{123}}$ be a size $N^4 \times 1$ vector that is generated as:

$$\boldsymbol{t_{123}} = \begin{bmatrix} t_1 vec(\boldsymbol{T_2}) \\ t_2 vec(\boldsymbol{T_2}) \\ \vdots \\ t_{N^2} vec(\boldsymbol{T_2}) \end{bmatrix} \qquad S6$$

where $t_i = (\boldsymbol{T_1} \otimes \boldsymbol{T}_3)[i,i]$. Then, Equation S4 can be written as:

$$vec(\boldsymbol{A_3}) = \boldsymbol{a_3} = \big(\boldsymbol{H_{d_1}^T} \otimes \boldsymbol{H_{d_4}}\big)\widehat{\boldsymbol{H}}_{\boldsymbol{d_{23}}}\boldsymbol{t_{123}} \qquad S7$$

where $\big(\boldsymbol{H_{d_1}^T} \otimes \boldsymbol{H_{d_4}}\big)\widehat{\boldsymbol{H}}_{\boldsymbol{d_{23}}}$ has rank N^2 since both $\boldsymbol{H_{d_1}^T} \otimes \boldsymbol{H_{d_4}}$ and $\widehat{\boldsymbol{H}}_{\boldsymbol{d_{23}}}$ have rank N^2.

There are also N^3-many nonzero elements of $\boldsymbol{t_{123}}$, each of which takes the form $t_{ijk} = t_{1,i}t_{2,j}t_{3,k}$ for $i,j,k \in \{1,2,\dots,N\}$. Therefore $\boldsymbol{a_3}$ can be written in the form:

$$\boldsymbol{a_3} = \sum_{i,j,k} t_{ijk}\boldsymbol{h_{ijk}} \qquad S8$$

where, $\boldsymbol{h_{ijk}}$ represents the corresponding column vector of $\big(\boldsymbol{H_{d_1}^T} \otimes \boldsymbol{H_{d_4}}\big)\widehat{\boldsymbol{H}}_{\boldsymbol{d_{23}}}$. Analogous to the 2-layer case, all t_{ijk} in Equation S8 ***cannot*** be chosen arbitrarily since it includes multiplicative terms of $t_{1,i}, t_{2,j}$ *and* $t_{3,k}$. However, by extending the algorithm detailed in Section S1 for the

current 3-layered network case, we can show that $\boldsymbol{a_3}$ can be formed as:

$$\boldsymbol{a_3} = \sum_{i=1}^{3N-2} c_i \boldsymbol{b_i} \tag{S9}$$

where c_i and $\boldsymbol{b_i}$ are generated by following an algorithm which is the counterpart of the layer and neuron selection algorithm presented for the 2-layer case in Table 1 of the main text. Based on this algorithm, we randomly select a diffractive layer and a neuron on that selected layer at each step of the algorithm to form c_i and $\boldsymbol{b_i}$ in Equation S9. We also present a special case of this algorithm in Table S2, where the layers are chosen in a regular, pre-determined order. By summing the used $\boldsymbol{h_{ijk}}$ vectors in the *5th* column of Table S2, it can be shown that N^3-many coefficients and vectors are used to generate the new coefficients. As a result, a set of $\boldsymbol{a_3}$ vectors can be generated from a $(3N-2)$-dimensional subspace of N^2-dimensional vector space. Also see Supplementary Section S4.3 and Supplementary Figure S3 for further analyses on *K*=3 case.

Step	Choice from $\boldsymbol{T_1}$	Choice from $\boldsymbol{T_2}$	Choice from $\boldsymbol{T_3}$	Resulting Coefficient and Basis Vector
1	$t_{1,1}$	$t_{2,1}$ (fixed)	$t_{3,1}$ (fixed)	$c_1\boldsymbol{b_1} = t_{1,1}\left(t_{2,1}t_{3,1}\boldsymbol{h_{111}}\right)$
2	-	-	$t_{3,2}$	$c_2\boldsymbol{b_2} = t_{3,2}\left(t_{1,1}t_{2,1}\boldsymbol{h_{112}}\right)$
3	-	$t_{2,2}$	-	$c_3\boldsymbol{b_3} = t_{2,2}\left(t_{1,1}t_{3,1}\boldsymbol{h_{121}} + t_{1,1}t_{3,2}\boldsymbol{h_{122}}\right)$
4	$t_{1,2}$	-	-	$c_4\boldsymbol{b_4} = t_{1,2}\left(t_{2,1}t_{3,1}\boldsymbol{h_{211}} + t_{2,1}t_{3,2}\boldsymbol{h_{212}} + t_{2,2}t_{3,1}\boldsymbol{h_{221}} + t_{2,2}t_{3,2}\boldsymbol{h_{222}}\right)$
5	-	-	$t_{3,3}$	$c_5\boldsymbol{b_5} = t_{3,3}\left(t_{1,1}t_{2,1}\boldsymbol{h_{113}} + t_{1,1}t_{2,2}\boldsymbol{h_{123}} + t_{1,2}t_{2,1}\boldsymbol{h_{213}} + t_{1,2}t_{2,2}\boldsymbol{h_{223}}\right)$
6	-	$t_{2,3}$	-	$c_6\boldsymbol{b_6} = t_{2,3}\left(t_{1,1}t_{3,1}\boldsymbol{h_{131}} + t_{1,1}t_{3,2}\boldsymbol{h_{132}} + t_{1,1}t_{3,3}\boldsymbol{h_{133}} + t_{1,2}t_{3,1}\boldsymbol{h_{231}} + +t_{1,2}t_{3,2}\boldsymbol{h_{232}} + t_{1,2}t_{3,3}\boldsymbol{h_{233}}\right)$
7	$t_{1,3}$	-	-	$c_7\boldsymbol{b_7} = t_{1,3}\left(t_{2,1}t_{3,1}\boldsymbol{h_{311}} + t_{2,1}t_{3,2}\boldsymbol{h_{312}} + t_{2,1}t_{3,3}\boldsymbol{h_{313}} + t_{2,2}t_{3,1}\boldsymbol{h_{321}} + t_{2,2}t_{3,2}\boldsymbol{h_{322}} + t_{2,2}t_{3,3}\boldsymbol{h_{323}} + t_{2,3}t_{3,1}\boldsymbol{h_{331}} + t_{2,3}t_{3,2}\boldsymbol{h_{332}} + t_{2,3}t_{3,3}\boldsymbol{h_{333}}\right)$
⋮	⋮	⋮	⋮	⋮
3n-4	-	-	$t_{3,n}$	$c_{3n-4}\boldsymbol{b_{3n-4}} = t_{3,n}\left(\sum_{i=1}^{n-1}\sum_{j=1}^{n-1} t_{1,i}t_{2,j}\boldsymbol{h_{ijn}}\right)$
3n-3	-	$t_{2,n}$	-	$c_{3n-3}\boldsymbol{b_{3n-3}} = t_{2,n}\left(\sum_{i=1}^{n-1}\sum_{k=1}^{n} t_{1,i}t_{3,k}\boldsymbol{h_{ink}}\right)$
3n-2	$t_{1,n}$	-	-	$c_{3n-2}\boldsymbol{b_{3n-2}} = t_{1,n}\left(\sum_{j=1}^{n}\sum_{k=1}^{n} t_{2,j}t_{3,k}\boldsymbol{h_{njk}}\right)$
⋮	⋮	⋮	⋮	⋮

3N-4	-	-	$t_{3,N}$	$c_{3N-4}\boldsymbol{b_{3N-4}} = t_{3,N}\left(\sum_{i=1}^{N-1}\sum_{j=1}^{N-1} t_{1,i}t_{2,j}\boldsymbol{h_{ijN}}\right)$
3N-3	-	$t_{2,N}$	-	$c_{3N-3}\boldsymbol{b_{3N-3}} = t_{2,N}\left(\sum_{i=1}^{N-1}\sum_{k=1}^{N} t_{1,i}t_{3,k}\boldsymbol{h_{iNk}}\right)$
3N-2	$t_{1,N}$	-	-	$c_{3N-2}\boldsymbol{b_{3N-2}} = t_{1,N}\left(\sum_{j=1}^{N}\sum_{k=1}^{N} t_{2,j}t_{3,k}\boldsymbol{h_{Njk}}\right)$

***Table S2**. Coefficient and basis vector generation algorithm for a 3-layered diffractive network (K = 3) when $N_{L1} = N_{L2} = N_{L3} = N_i = N_o = N$. Note that the regular selection order of the diffractive layers presented in this table is a special case, and is different from the algorithm that we used in generating the rank values reported in Fig. S3. This special case presents a pre-determined selection order and does not necessarily represent the optimum order of diffractive layer and neuron selection. For the rank values reported in Fig. S3, we randomly select a diffractive layer and a neuron on that selected layer at each step of the algorithm (see Supplementary Section S2), which forms an extended version of the algorithm presented in Table 1 of the main text.*

S3. Coefficient and basis vector generation algorithm for an optical network formed by K diffractive surfaces: Special case for $N_{L1} = N_{L2} = \cdots = N_{LK} = N_i = N_o = N$

In the subsection of the main text titled "*Analysis of an optical network formed by three or more diffractive surfaces*", it was shown that each additional diffractive surface that has size *N* increases the dimensionality of the resulting transformation vector by $N - 1$, unless the diffractive network reaches its capacity for a given pair of input-output fields-of-view. The previous two sections (S1 and S2) have also confirmed the same conclusion. In this section, we independently confirm this statement by representing a special case of the algorithm presented in Table 1 of the main text.

Let us assume a diffractive network that has the dimensionality of the optical solution space as $(K - 1)N - (K - 2)$. This dimensionality can potentially be achieved by a network that has $K - 1$ many independent diffractive layers where each surface has N trainable neurons or through only one diffractive layer that has $(K - 1)N - (K - 2)$ neurons. Here, we assume that there is initially a single diffractive surface that has $N_{L1} = (K - 1)N - (K - 2)$ trainable neurons and then a second diffractive surface that has $N_{L2} = N$ neurons is added to this diffractive network. Based on this assumption, we can rewrite Equations 3 and 4 of the main text as:

$$\boldsymbol{y} = \boldsymbol{H}'_{\boldsymbol{d_3}}\boldsymbol{T_2}\boldsymbol{H_{d_2}}\boldsymbol{T_1}\boldsymbol{H}'_{\boldsymbol{d_1}}\boldsymbol{x} = \boldsymbol{A_2}\boldsymbol{x} \qquad S10$$

and

$$vec(\boldsymbol{A_2}) = \boldsymbol{a_2} = \left(\boldsymbol{H}'^{T}_{\boldsymbol{d_1}} \otimes \boldsymbol{H}'_{\boldsymbol{d_3}}\right)\widehat{\boldsymbol{H}}_{\boldsymbol{d_2}}\boldsymbol{t_{12}} \qquad S11$$

where, in this case, $N_{L1} = (K-1)N - (K-2)$ and $N_{L2} = N$. Therefore, $\boldsymbol{a_2}$ can be written as:

$$\boldsymbol{a_2} = \sum_{i,j} t_{ij}\boldsymbol{h_{ij}} \quad , \qquad S12$$

where $\boldsymbol{h_{ij}}$ is the corresponding column vector of $\left(\boldsymbol{H}'^{T}_{\boldsymbol{d_1}} \otimes \boldsymbol{H}'_{\boldsymbol{d_3}}\right)\widehat{\boldsymbol{H}}_{\boldsymbol{d_2}}$ which has rank N^2 and $t_{ij} = t_{1,i}t_{2,j}$, where $t_{1,i}$ and $t_{2,j}$ are the trainable complex-valued transmission coefficients of the ith neuron of the 1st diffractive surface and the jth neuron of the 2nd diffractive surface, respectively, for $i \in \{1,2,\dots,(K-1)N-(K-2)\}$ and $j \in \{1,2,\dots,N\}$. Hence, we can show that $\boldsymbol{a_2}$ can be written as:

$$\boldsymbol{a_2} = \sum_{i=1}^{KN-(K-1)} c_i\boldsymbol{b_i} \quad , \qquad S13$$

where c_i and $\boldsymbol{b_i}$ are an arbitrary complex-valued coefficient and the corresponding basis vector, respectively. The algorithm that we use here is a special case of the algorithm given in the subsection of the main text titled "*Coefficient and basis vector generation for an optical network formed by two diffractive surfaces*" for the chunk partition $n_1 = n_3 = \cdots = n_{2i-1} = \cdots = n_{s-1} = 1$ and $n_2 = n_4 = \cdots = n_{2i} = \cdots = n_s = K$. That is, after choosing one coefficient from $t_{1,i}$ and $t_{2,j}$ at the initialization step, we choose one coefficient from $t_{2,j}$ in the second step and one coefficient from $t_{1,i}$ in each of the following K steps. In each step, we use the previously chosen coefficients and the corresponding $\boldsymbol{h_{ij}}$ vectors in order to create the next basis vector. Then, this procedure continues until all the coefficients have been used.

We summarized these steps of the coefficient and basis generation algorithm in Table S3, in the next page. It can be shown that, all $N[(K-1)N-(K-2)]$ nonzero coefficients (t_{ij}'s) are used to generate $KN-(K-1)$ basis vectors. This analysis confirms that if there are $(K-1)N-(K-2)$ neurons in a single layered network, the addition of a second diffractive layer with N independent neurons can expand the dimensionality of the possible input-output transformations by $N-1$, assuming the capacity limit (N^2) has not been reached.

Step	Choice from $\boldsymbol{T_1}$	Choice from $\boldsymbol{T}_2$	Resulting Coefficient and Basis Vector
1	$t_{1,1}$	$t_{2,1}$ (fixed)	$c_1\boldsymbol{b_1} = t_{1,1}\left(t_{2,1}\boldsymbol{h_{11}}\right)$
2	-	$t_{2,2}$	$c_2\boldsymbol{b_2} = t_{2,2}\left(t_{1,1}\boldsymbol{h_{12}}\right)$
3	$t_{1,2}$	-	$c_{i+1}\boldsymbol{b_{i+1}} = t_{1,i}\left(t_{2,1}\boldsymbol{h_{i1}} + t_{2,2}\boldsymbol{h_{i2}}\right)$, for $i \in \{2,3,\dots,K\}$
4	$t_{1,3}$	-	
$\vdots$	$\vdots$	$\vdots$	
K+1	$t_{1,K}$	-	
K+2	-	$t_{2,3}$	$c_{K+2}\boldsymbol{b}_{K+\boldsymbol{2}} = t_{2,3}\left(\sum_{i=2}^{K} t_{1,i}\boldsymbol{h_{i1}}\right)$
K+3	$t_{1,K+1}$	-	$c_{i+2}\boldsymbol{b_{i+2}} = t_{1,i}\left(t_{2,1}\boldsymbol{h_{i1}} + t_{2,2}\boldsymbol{h_{i2}} + t_{2,3}\boldsymbol{h_{i3}}\right)$, for $i \in \{K+1, K+2, \dots, 2K-1\}$
K+4	$t_{1,K+2}$	-	
$\vdots$	$\vdots$	$\vdots$	
2K+1	$t_{1,2K-1}$	-	
$\vdots$	$\vdots$	$\vdots$	$\vdots$
qK+2	-	$t_{2,q+2}$	$c_{qK+2}\boldsymbol{b_{qK+2}} = t_{2,q+2}\left(\sum_{i=1}^{qK-(q-1)} t_{1,i}\boldsymbol{h_{i(q+2)}}\right)$
qK+3	$t_{1,q(K-1)+2}$	-	$c_{i+1}\boldsymbol{b_{i+1}} = t_{1,i-q}\left(\sum_{j=1}^{q+2} t_{2,j}\boldsymbol{h_{(i-q)j}}\right)$, for $i \in \{qK+2, qK+3, \dots, qK+K\}$
qK+4	$t_{1,q(K-1)+3}$	-	
$\vdots$	$\vdots$	$\vdots$	
(q+1)K+1	$t_{1,(q+1)K-q}$	-	
$\vdots$	$\vdots$	$\vdots$	$\vdots$
(N-2)K+2	-	$t_{2,N}$	$c_{(N-2)K+2}\boldsymbol{b_{(N-2)K+2}} = t_{2,N}\left(\sum_{i=1}^{(N-2)K-(N-1)} t_{1,i}\boldsymbol{h_{iN}}\right)$
(N-2)K+3	$t_{1,(N-2)(K-1)+2}$	-	$c_{i+N-1}\boldsymbol{b_{i+N-1}} = t_{1,i}\left(\sum_{j=1}^{N} t_{2,j}\boldsymbol{h_{ij}}\right)$, for $i \in \{(N-2)(K-1)+2, (N-2)(K-1)+3, \dots, (N-1)K-(N-2)\}$
(N-2)K+4	$t_{1,(N-2)(K-1)+3}$	-	
$\vdots$	$\vdots$	$\vdots$	
KN-(K-1)	$t_{1,(N-1)K-(N-2)}$	-	

Table S3. *Coefficient and basis vector generation algorithm for a 2-layered diffractive network when $N_{L1} = (K-1)N - (K-2)$ and $N_{L2} = N_i = N_o = N$*

S4. Computation of the dimensionality (D) of the all-optical solution space for K = 1, 2 and 3

In order to calculate D for various diffractive network configurations, we used the symbolic toolbox of MATLAB to compute the rank of diffraction related matrices using their symbolic representation.

S4.1. 1-Layer Case (K = 1):

To compute D for $K = 1$, we first generate $\boldsymbol{H}'^{T}_{\boldsymbol{d_1}} \otimes \boldsymbol{H}'_{\boldsymbol{d_2}}$ of Equation 2 (main text). Note that for $K = 1$ only N_{L1} −many columns of $\boldsymbol{H}'^{T}_{\boldsymbol{d_1}} \otimes \boldsymbol{H}'_{\boldsymbol{d_2}}$ are included in the computation of $vec(\boldsymbol{A_1})$. Therefore, we consider only those vectors in our computation. We define $\boldsymbol{H}'$ as the matrix which is subject to the rank computation:

$$\boldsymbol{H}'[:,m] = (\boldsymbol{H}'^{T}_{\boldsymbol{d_1}} \otimes \boldsymbol{H}'_{\boldsymbol{d_2}})[:,m(N_{L1}+1)] \qquad S14$$

for $m \in \{0,1,\dots,N_{L1}-1\}$. Here, $[:,m(N_{L1}+1)]$ indicates the column associated with the $(m+1)^{th}$ neuron in the vectorized form. Hence, in 2D discrete space, m corresponds to a certain neuron position and discrete index, $[q_{L1}, p_{L1}]$.

$\boldsymbol{H}'[l,m]$ takes its values through the multiplication of the appropriate free space impulse response functions from the associated input pixel (within N_i) to the $(m+1)^{th}$ neuron and from the $(m+1)^{th}$ neuron to the associated output pixel (within N_o). Thus, a given l corresponds to a certain position at the input plane, $[q_i, p_i]$, paired with a certain position at the output plane, $[q_o, p_o]$. As a result, $\boldsymbol{H}'[l,m]$ can be written as:

$$\boldsymbol{H}'[l,m] = h_{d1}(q_i - q_{L1}, p_i - p_{L1}) \cdot h_{d2}(q_o - q_{L1}, p_o - p_{L1}) \quad , \qquad S15$$

where $d_1 \neq d_2 \neq 0$ and $h_d(x,y)$ is the impulse response of free space propagation, which can be written as:

$$h_d(x,y) = -\frac{e^{j\frac{2\pi}{\lambda}r}}{2\pi}\left(j\frac{2\pi}{\lambda} - \frac{1}{r}\right)\frac{d}{r^2} \quad , \qquad S16$$

where $r = \sqrt{x^2 + y^2 + d^2}$.

In MATLAB, we used various symbolic conversion schemes to confirm that each method ends up with the same rank. For a given $N_i = N_o = N_{FOV}$, N_{L1}, d_1 and d_2 configuration, in the first four methods, we generated $\boldsymbol{H}'$ numerically in the double precision. Then we converted it to the corresponding symbolic matrix representation using either one of these commands:

>>*sym*($\boldsymbol{H}'$,'r') *(Method 1.a)*

>>*sym*($\boldsymbol{H}'$,'d') *(Method 1.b)*

>>*sym*($\boldsymbol{H}'$,'e') *(Method 1.c)*

>>*sym*($\boldsymbol{H}'$,'f') *(Method 1.d)*

In the second set of symbolic conversion schemes, in order to further increase the precision in our computation, we generated π symbolically at the beginning as:

>>*sym(pi,'r')* *(Method 2.a)*

>>*sym(pi,'d')* *(Method 2.b)*

>>*sym(pi,'e')* *(Method 2.c)*

>>*sym(pi,'f')* *(Method 2.d)*

Then we generated $\boldsymbol{H'}$ of Equation S15 using the symbolic π, which ended up with a symbolic $\boldsymbol{H'}$ matrix. Note that, although the second set of methods has a better accuracy in symbolic representation, they require more computation memory and time in generating the rank result. So, in our rank computations, we used *Method 1.a* as the common method for all the diffractive network configurations reported in Supplementary Figures S1-S3. Besides *Method 1.a*, we also used at least one of the remaining seven methods in each diffractive network configuration to confirm that the resulting rank values agree with each other.

Figure S1 summarizes the resulting rank calculations for various different K = 1 diffractive network configurations, all of which confirm $D = min(\ N_{L1}\ , N_{FOV}^2\ )$. $D = N_{L1}$ results reported in Figure S1 indicate that all the columns of $\boldsymbol{H'}$ are linearly independent, and therefore any subset of its columns are also linearly independent. This shows that the dimensionality of the solution space for $d_1 \neq d_2$ is a linear function of N_{L1} when $N_{L1} \leq N_{FOV}^2$, and N_{FOV}^2 defines the upper limit of D (also see Figure 2 of the main text). In Supplementary Section S5, we also show that the upper limit for the dimensionality of the all-optical solution space reduces to $N_{FOV}(N_{FOV}+1)/2$ when $d_1 = d_2$ for a single diffractive layer, $K = 1$.

S4.2. 2-Layer Case (*K* = 2):

For $K = 2$, we deal with the matrix $\left(\boldsymbol{H}_{\boldsymbol{d_1}}^{\prime T} \otimes \boldsymbol{H}'_{\boldsymbol{d_3}}\right)\widehat{\boldsymbol{H}}_{\boldsymbol{d_2}}$ of Equation 4 of the main text. We first generated a matrix $\boldsymbol{H'}$ from $\left(\boldsymbol{H}_{\boldsymbol{d_1}}^{\prime T} \otimes \boldsymbol{H}'_{\boldsymbol{d_3}}\right)\widehat{\boldsymbol{H}}_{\boldsymbol{d_2}}$ such that the columns of $\left(\boldsymbol{H}_{\boldsymbol{d_1}}^{\prime T} \otimes \boldsymbol{H}'_{\boldsymbol{d_3}}\right)\widehat{\boldsymbol{H}}_{\boldsymbol{d_2}}$ that correspond to the zero entries of $\boldsymbol{t_{12}}$ are discarded. First, we converted $\boldsymbol{H'}$ into a symbolic matrix and then applied the algorithm presented in Table 1 of the main text on the columns of $\boldsymbol{H'}$. Here the mth column of $\boldsymbol{H'}$ is the vector that multiplies the coefficient $t_{1,i}t_{2,j}$ of $\boldsymbol{t_{12}}$ of Equation 4 of the main text for a certain (i,j) pair, i.e., there is a one-to-one relationship between a given m and the associated (i,j) pair.

Therefore, a given m indicates a certain neuron position in the first diffractive layer, $[q_{L1}, p_{L1}]$, paired with a certain neuron position in the second diffractive layer, $[q_{L2}, p_{L2}]$. Similar to the K = 1 case, the lth row of $\boldsymbol{H'}$ corresponds to a certain set of input and output pixels as part of N_i and N_o, respectively, and $\boldsymbol{H'}[l,m]$ can be written as:

$$\boldsymbol{H'}[l,m] = h_{d1}(q_i - q_{L1}, p_i - p_{L1}) \cdot\ h_{d3}(q_o - q_{L2}, p_o - p_{L2}) \cdot\ h_{d2}(q_{L1} - q_{L2}, p_{L1} - p_{L2}) \quad S17$$

After generating $\boldsymbol{H'}$ based on Equation S17, we converted it into the symbolic matrix representation as described earlier for the 1-layer case, K = 1. Then, we applied the algorithm presented in Table 1 of the main text to generate the basis vectors and their coefficients. Note that, for each diffractive network configuration that we selected, we independently ran the same algorithm three times with different random initializations, random selection of the neurons and random generation of complex-valued transmission coefficients. In all of the rank results that are reported in Figure S2, these repeated simulations agreed with each other and gave the same rank, confirming $D = min(N_{L1} + N_{L2} - 1, N_{FOV}^2)$. Also note that, unlike the $d_1 = d_2$ case for $K = 1$ (Section S5), different combinations of d_1, d_2 and d_3 values for K = 2 do not change the results or the upper bound of D, as also confirmed in Figure S2.

S4.3. 3-Layer Case (*K* = 3):

For K = 3 case, we start with $\left(\boldsymbol{H}_{\boldsymbol{d_1}}^T \otimes \boldsymbol{H}_{\boldsymbol{d_4}}\right)\widehat{\boldsymbol{H}}_{\boldsymbol{d_{23}}}$ of Equation S7. Then, we generate the matrix $\boldsymbol{H'}$ by discarding the columns of $\left(\boldsymbol{H}_{\boldsymbol{d_1}}^T \otimes \boldsymbol{H}_{\boldsymbol{d_4}}\right)\widehat{\boldsymbol{H}}_{\boldsymbol{d_{23}}}$ that correspond to the zero entries of $\boldsymbol{t_{123}}$ of Equation S7. Here, the mth column of $\boldsymbol{H'}$ is the vector that multiplies the coefficient $t_{1,i}t_{2,j}t_{3,k}$ for a certain (i, j, k) triplet. Hence, there is a one-to-one relationship between a given m and the pixel/neuron locations from the first, second and third diffractive layers, which are represented by $[q_{L1}, p_{L1}]$, $[q_{L2}, p_{L2}]$ and $[q_{L3}, p_{L3}]$, respectively. Similar to the K = 1 and K = 2 cases discussed in earlier sections, a given row, l, corresponds to a certain set of input (from N_i) and output (from N_o) pixels, $[q_i, p_i]$ and $[q_o, p_o]$, respectively. Accordingly, $\boldsymbol{H'}[l, m]$ can be written as:

$$\begin{aligned}\boldsymbol{H'}[l,m] &= h_{d1}(q_i - q_{L1}, p_i - p_{L1}) \cdot h_{d4}(q_o - q_{L3}, p_o - p_{L3}) \\ &\cdot h_{d2}(q_{L1} - q_{L2}, p_{L1} - p_{L2}) \cdot h_{d3}(q_{L2} - q_{L3}, p_{L2} - p_{L3})\end{aligned} \quad S18$$

Then, we applied a coefficient and basis generation algorithm that is similar to Table 1 of the main text, where we randomly select the diffractive layer and the neuron in each step of the algorithm to obtain the resulting coefficients and the basis vectors. Then we converted the resulting vectors into their symbolic representations as discussed earlier for the K = 1 case and computed the rank of the resulting symbolic matrix. For K = 3 the selection order of the 1st, 2nd and 3rd diffractive layers in consecutive steps and the location/value of the chosen neuron at each step may affect the computed rank. Especially, when $N_{L1} + N_{L2} + N_{L3}$ is close to N_{FOV}^2, the probability of repeatedly achieving the upper-bound of the dimensionality of the solution space, i.e., $min(N_{L1} + N_{L2} + N_{L3} - 2, N_{FOV}^2)$, using random orders of selection decreases. On the other hand, when $N_{L1} + N_{L2} + N_{L3}$ is considerably larger than N_{FOV}^2, due to the additional degrees of freedom in the diffractive system, the probability of repeatedly achieving the upper-bound of the dimensionality using random orders of selection significantly increases. In Figure S3, we present the computed ranks for different K=3 diffractive network configurations; for each one of these configurations that we considered in our simulations, we obtained at least one random selection of the diffractive layers and neurons that attained full rank, numerically confirming $D = min(N_{L1} + N_{L2} + N_{L3} - 2, N_{FOV}^2)$.

S5. The Upper Bound of the Dimensionality (D) of the Solution Space Reduces to $\left(N_{FOV}^2 + N_{FOV}\right)/2$ when $d_1 = d_2$ for $K = 1$

For K = 1 and the special case of $d_1 = d_2 = d$, we can rewrite $\boldsymbol{H}'[l, m]$ given by Equation S15 as:

$$\boldsymbol{H}''[l, m] = h_d(q_i - q_{L1}, p_i - p_{L1})\, h_d(q_o - q_{L1}, p_o - p_{L1}). \qquad S19$$

To quantify the reduction in rank due to $d_1 = d_2$, among N_{FOV}^2 entries of $\boldsymbol{H}''[:, m]$, let us first consider the cases where $(q_i, p_i) \neq (q_o, p_o)$. For a given neuron or m, assuming that $(q_i, p_i) \neq (q_o, p_o)$, the number of different entries that can be produced by Equation S19 becomes $C\binom{N_{FOV}}{2}$, where $C\binom{\cdot}{\cdot}$ indicates the combination operation and $N_{FOV} = N_i = N_o$. Stated differently, since $h_{d1} = h_{d2}$ the order of the selections from (q_i, p_i) and (q_o, p_o) does not matter, making the selection defined by a combination operation, i.e., $C\binom{N_{FOV}}{2}$. In addition to these combinatorial entries, there are N_{FOV} additional entries that represent $(q_i, p_i) = (q_o, p_o)$. Therefore, the total number of unique entries in a column, $\boldsymbol{H}''[:, m]$, becomes:

$$C\binom{N_{FOV}}{2} + N_{FOV} = (N_{FOV}^2 + N_{FOV})/2. \qquad S20$$

This analysis proves that, for K = 1, the upper limit of the dimensionality (D) of the all-optical solution space for $d_1 = d_2$ reduces from N_{FOV}^2 to $(N_{FOV}^2 + N_{FOV})/2$ due to the fact that $h_{d1} = h_{d2} = h_d$ in Equation S19.

Note that, when $d_1 \neq d_2$, we have $h_{d1} \neq h_{d2}$, which directly implies that the combination operation in Equation S20 must be replaced with the permutation operation, $P\binom{\cdot}{\cdot}$, since the order of selections from (q_i, p_i) and (q_o, p_o) matters (see Equation S15). Therefore, when $d_1 \neq d_2$, Equation S20 is replaced with:

$$P\binom{N_{FOV}}{2} + N_{FOV} = N_{FOV}^2 \qquad S21$$

which confirms our analyses in the main text, reported in the subsection "*Analysis of a single diffractive surface*" as well as the results reported in Figure S1.

(a) K=1, $N_i = N_o = N_{FOV} = 4 \times 4$

$D = min(N_{L1}, N_i \times N_o)$	$N_{L1} = N_{y1} \times N_{x1}$						
	20 x 20	16 x 16	12 x 12	12 x 11	11 x 12	17 x 8	8 x 17
$d_1 = \lambda$, $d_2 = 4\lambda$	256	256	144	132	132	136	136
$d_1 = 4\lambda$, $d_2 = \lambda$	256	256	144	132	132	136	136
$d_1 = 4\lambda$, $d_2 = 64\lambda$	256	256	144	132	132	136	136
$d_1 = 64\lambda$, $d_2 = 4\lambda$	256	256	144	132	132	136	136
$d_1 = 1024\lambda$, $d_2 = 16\lambda$	256	256	144	132	132	136	136
$d_1 = 16\lambda$, $d_2 = 1024\lambda$	256	256	144	132	132	136	136
$d_1 = 64\lambda$, $d_2 = 1\lambda$	256	256	144	132	132	136	136
$d_1 = 1\lambda$, $d_2 = 64\lambda$	256	256	144	132	132	136	136

(b) K=1, $N_i = N_o = N_{FOV} = 8 \times 8$

$D = min(N_{L1}, N_i \times N_o)$	D
$d_1 = 1\lambda$, $d_2 = 4\lambda$, $N_{L1} = 64 \times 64$	4096
$d_1 = 4\lambda$, $d_2 = 64\lambda$, $N_{L1} = 64 \times 64$	4096
$d_1 = 1024\lambda$, $d_2 = 16\lambda$, $N_{L1} = 64 \times 64$	4096
$d_1 = 64\lambda$, $d_2 = 1\lambda$, $N_{L1} = 64 \times 64$	4096
$d_1 = 1\lambda$, $d_2 = 4\lambda$, $N_{L1} = 46 \times 46$	2116
$d_1 = 4\lambda$, $d_2 = 64\lambda$, $N_{L1} = 46 \times 46$	2116
$d_1 = 1024\lambda$, $d_2 = 16\lambda$, $N_{L1} = 46 \times 46$	2116
$d_1 = 64\lambda$, $d_2 = 1\lambda$, $N_{L1} = 46 \times 46$	2116
$d_1 = 4\lambda$, $d_2 = 64\lambda$, $N_{L1} = 40 \times 52$	2080
$d_1 = 4\lambda$, $d_2 = 64\lambda$, $N_{L1} = 52 \times 40$	2080
$d_1 = 4\lambda$, $d_2 = 64\lambda$, $N_{L1} = 20 \times 104$	2080
$d_1 = 4\lambda$, $d_2 = 64\lambda$, $N_{L1} = 104 \times 20$	2080
$d_1 = 1\lambda$, $d_2 = 4\lambda$, $N_{L1} = 32 \times 128$	4096
$d_1 = 1\lambda$, $d_2 = 4\lambda$, $N_{L1} = 128 \times 32$	4096

Figure S1: Computation of the dimensionality (D) of the all-optical solution space for K=1 diffractive surface under various network configurations. The rank values are obtained using the symbolic toolbox of MATLAB from $\boldsymbol{H}'$ matrix, using Equation S15. The calculated rank values in each table obey the rule $D = min(\, N_{L1}\, , N_{FOV}^2\,)$. $D = N_{L1}$ results indicate that all the columns of $\boldsymbol{H}'$ are linearly independent, and therefore any subset of its columns are also linearly independent. Therefore, the dimensionality of the solution space for $d_1 \neq d_2$ is a linear function of N_{L1} when $N_{L1} \leq N_{FOV}^2$, and N_{FOV}^2 defines the upper limit of D (see Figure 2 of the main text). In Supplementary Section S5, we also show that the upper limit for the dimensionality of the all-optical solution space reduces to $N_{FOV}(N_{FOV} + 1)/2$ when $d_1 = d_2$ for a single diffractive layer, $K = 1$.

(a) K=2, $N_i = N_o = N_{FOV}$ = 4 x 4

$D = min(N_{L1} + N_{L2} - 1, N_i \times N_o)$	$N_{L1} = N_{y1} \times N_{x1}$ $N_{L2} = N_{y2} \times N_{x2}$						
	N_{L1} = 16 x 10, N_{L2} = 10 x 16	N_{L1} = 16 x 8, N_{L2} = 8 x 16	N_{L1} = 14 x 14, N_{L2} = 7 x 7	N_{L1} = 7 x 7, N_{L2} = 14 x 14	N_{L1} = 8 x 8, N_{L2} = 10 x 10	N_{L1} = 10 x 10, N_{L2} = 8 x 8	N_{L1} = 11 x 11, N_{L2} = 11 x 11
$d_1 = d_2 = d_3 = \lambda$	256	255	244	244	163	163	241
$d_1 = d_2 = d_3 = 4\lambda$	256	255	244	244	163	163	241
$d_1 = \lambda$, $d_2 = 4\lambda$, $d_3 = 16\lambda$	256	255	244	244	163	163	241
$d_1 = d_2 = d_3 = 10^3 \lambda$	256	255	244	244	163	163	241
$d_1 = d_2 = d_3 = 4 \times 10^3 \lambda$	256	255	244	244	163	163	241
$d_1 = 10^3 \lambda$, $d_2 = 4 \times 10^3 \lambda$, $d_3 = 16 \times 10^3 \lambda$	256	255	244	244	163	163	241

(b) K=2, $N_i = N_o = N_{FOV}$ = 6 x 6 and 7 x 7

$D = min(N_{L1} + N_{L2} - 1, N_i \times N_o)$		D
$N_i = N_o$ = 6 x 6, N_{L1} = 36 x 18, N_{L2} = 18 x 36	$d_1 = d_2 = d_3 = \lambda$	1295
	$d_1 = d_2 = d_3 = 4\lambda$	1295
	$d_1 = 1\lambda$, $d_2 = 4\lambda$, $d_3 = 16\lambda$	1295
	$d_1 = d_2 = d_3 = 10^3 \lambda$	1295
	$d_1 = d_2 = d_3 = 4 \times 10^3 \lambda$	1295
	$d_1 = 10^3 \lambda$, $d_2 = 4 \times 10^3 \lambda$, $d_3 = 16 \times 10^3 \lambda$	1295
$N_i = N_o$ = 7 x 7, N_{L1} = 32 x 48, N_{L2} = 48 x 32	$d_1 = d_2 = d_3 = \lambda$	2401
	$d_1 = d_2 = d_3 = 4\lambda$	2401
	$d_1 = 1\lambda$, $d_2 = 4\lambda$, $d_3 = 16\lambda$	2401
	$d_1 = d_2 = d_3 = 10^3 \lambda$	2401
	$d_1 = d_2 = d_3 = 4 \times 10^3 \lambda$	2401
	$d_1 = 10^3 \lambda$, $d_2 = 4 \times 10^3 \lambda$, $d_3 = 16 \times 10^3 \lambda$	2401

(c) K=2, $N_i = N_o = N_{FOV}$ = 8 x 8

$D = min(N_{L1} + N_{L2} - 1, N_i \times N_o)$	$N_{L1} = N_{y1} \times N_{x1}$ $N_{L2} = N_{y2} \times N_{x2}$	
	N_{L1} = 24 x 48, N_{L2} = 48 x 24	N_{L1} = 24 x 24, N_{L2} = 12 x 12
$d_1 = d_2 = d_3 = \lambda$	2303	719
$d_1 = d_2 = d_3 = 4\lambda$	2303	719
$d_1 = 1\lambda$, $d_2 = 4\lambda$, $d_3 = 16\lambda$	2303	719
$d_1 = d_2 = d_3 = 10^3 \lambda$	2303	719
$d_1 = d_2 = d_3 = 4 \times 10^3 \lambda$	2303	719
$d_1 = 10^3 \lambda$, $d_2 = 4 \times 10^3 \lambda$, $d_3 = 16 \times 10^3 \lambda$	2303	719

Figure S2: Computation of the dimensionality (*D*) of the all-optical solution space for *K*=2 diffractive surfaces under various network configurations. The rank values are obtained using the symbolic toolbox of MATLAB from $\boldsymbol{H'}$ matrix, based on Equation S17 and Table 1 of the main text. Each result in the presented tables is confirmed through three independent runs of the same algorithm with different random initializations, random selection of the neurons and random generation of complex-valued transmission coefficients. All the presented rank results numerically confirm $D = min(\,N_{L1} + N_{L2} - 1\,, N_{FOV}^2)$.

K=3, $N_i = N_o = N_{FOV} = 4 \times 4$

$D = min(N_{L1} + N_{L2} + N_{L3} - 2, N_i \times N_o)$	$N_{L1} = N_{y1} \times N_{x1}$ $N_{L2} = N_{y2} \times N_{x2}$ $N_{L3} = N_{y3} \times N_{x3}$	
	$N_{L1} = 6 \times 12$ $N_{L2} = 18 \times 6$ $N_{L3} = 18 \times 12$	$N_{L1} = 16 \times 8$ $N_{L2} = 8 \times 6$ $N_{L3} = 8 \times 10$
$d_1 = d_2 = d_3 = d_4 = \lambda$	256	254
$d_1 = d_2 = d_3 = d_4 = 4\lambda$	256	254
$d_1 = \lambda$, $d_2 = 4\lambda$, $d_3 = 8\lambda$, $d_4 = 16\lambda$	256	254
$d_1 = d_2 = d_3 = d_4 = 10^3 \lambda$	256	254
$d_1 = d_2 = d_3 = d_4 = 4 \times 10^3 \lambda$	256	254
$d_1 = 10^3 \lambda$, $d_2 = 4 \times 10^3 \lambda$, $d_3 = 8 \times 10^3 \lambda$, $d_4 = 16 \times 10^3 \lambda$	256	254

Figure S3: Computation of the dimensionality (*D*) of the all-optical solution space for *K*=3 diffractive surfaces under various network configurations. The rank values are obtained using the symbolic toolbox of MATLAB from $\boldsymbol{H'}$ matrix, based on Equation S18 and the algorithm discussed in Section S2 (also see the supplementary text, Section S4.3, for details). The presented results indicate that it is possible to obtain the maximum dimensionality of the solution space, numerically confirming that $D = min(N_{L1} + N_{L2} + N_{L3} - 2, N_{FOV}^2)$.

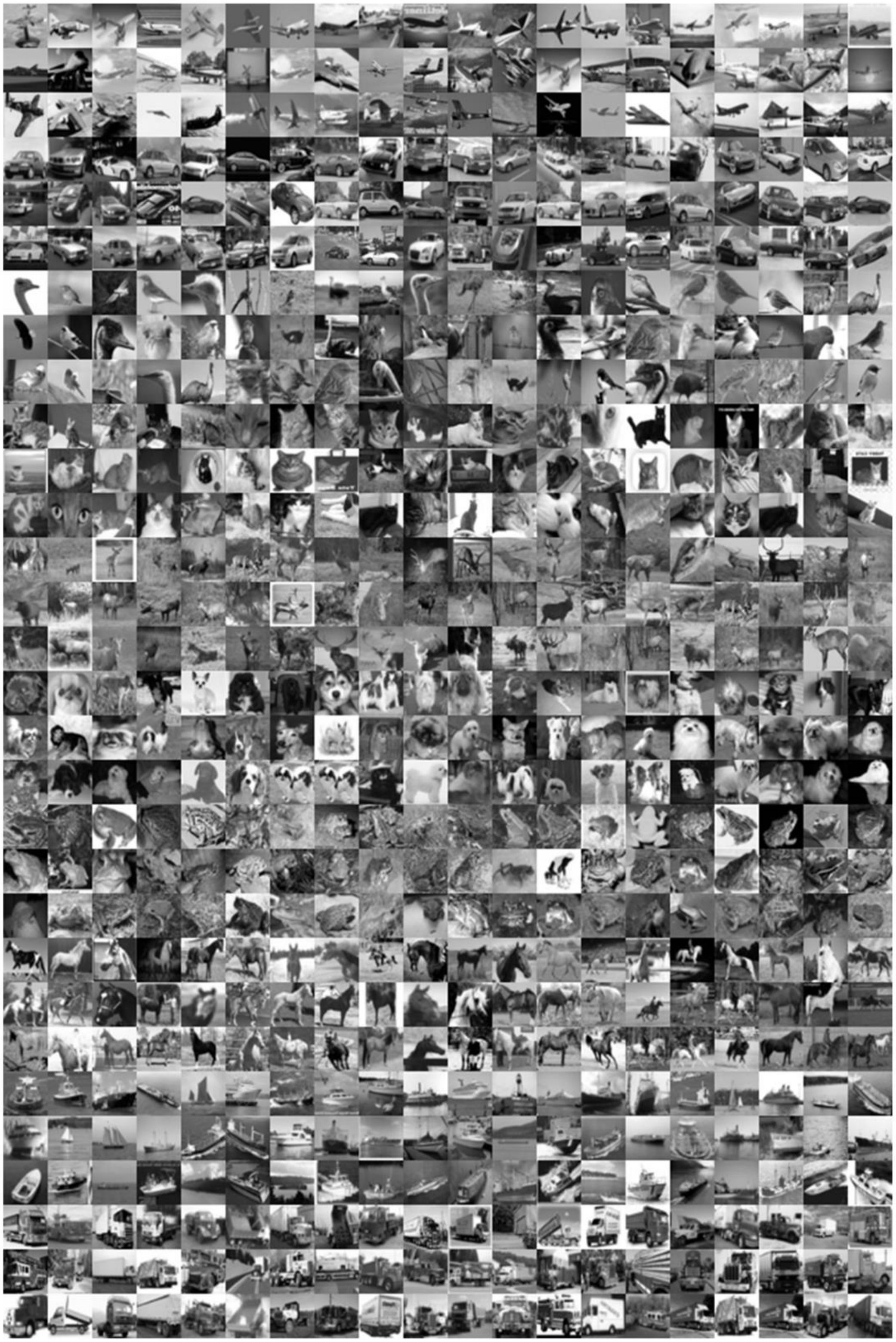

Figure S4: Randomly selected samples from the 50K training set in the original CIFAR-10 dataset. The images are converted from RGB to gray-scale by selecting the Y-channel in the corresponding YCrCb representations. There are in total 600 samples (60 per data class) depicted here. Every 3 rows contain the samples from a data class resulting in a total of 30 rows.

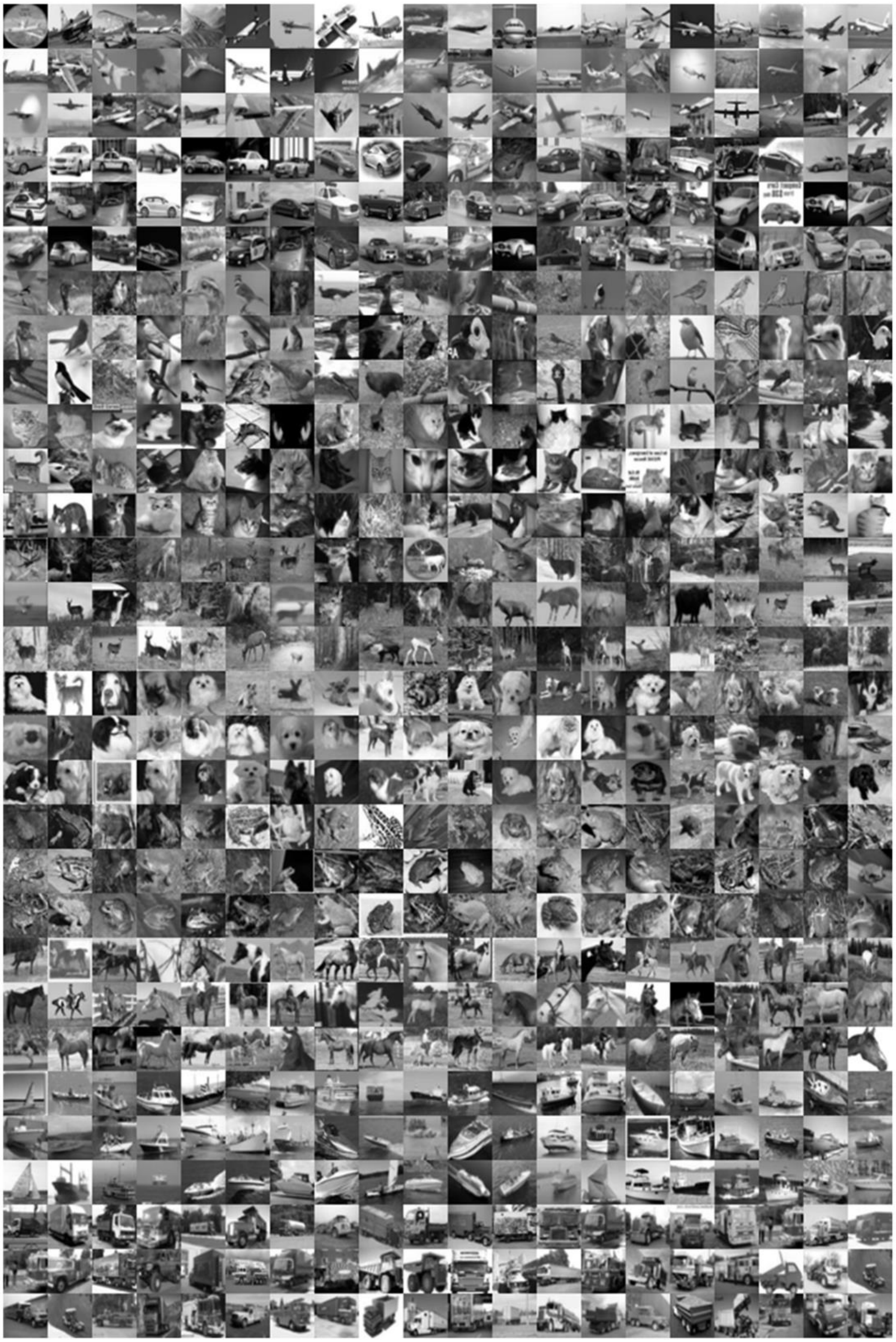

Figure S5: Randomly selected samples from the 10K testing set in the original CIFAR-10 dataset. The images are converted from RGB to gray-scale by selecting the Y-channel in the corresponding YCrCb representations. There are in total 600 samples (60 per data class) depicted here. Every 3 rows contain the samples from a data class resulting in a total of 30 rows.
